# Prevalence and screening of interstitial lung disease in rheumatoid arthritis

**DOI:** 10.1093/rap/rkag062

**Published:** 2026-06-03

**Authors:** Takahisa Gono

**Affiliations:** ^1^ Department of Allergy and Rheumatology, Nippon Medical School Graduate School of Medicine, Tokyo, Japan

**This editorial refers to: ‘The prevalence of interstitial lung disease in rheumatoid arthritis: a systematic review’ by Vermant et al. 2026, doi: 10.1093/rap/rkag045** 

In Marie Vermant et al.’s article [1] in this issue of Rheumatology Advances in Practice, a systematic literature review (SLR) and meta-analysis have been conducted to assess the prevalence of RA-associated interstitial lung disease (RA-ILD). This SLR included 74 studies and estimated an overall random-effect pooled prevalence of 20.6% (95% CI, 18.3–23.2%, *I*^2^ = 99.7%). The random-effects pooled prevalence varied by diagnostic method: 31% (95% CI, 25–38%, *I*^2^ = 97%) when using high-resolution CT (HRCT), 21% (95% CI, 14–31%, *I*^2^ = 95%) with pulmonary function tests (PFTs), 13% (95% CI, 5–30%, *I*^2^ = 99%) with combining multiple diagnostic tools, including chest X-ray, PFTs, and HRCT, 12% (95% CI, 7–20%, *I*^2^ = 96%) with chest X-ray, and 5% (95% CI, 4–8%, *I*^2^ = 100%) when based on International Classification of Diseases codes. These markedly high levels of heterogeneity typically indicate significant clinical and/or methodological disparities, such as different screening triggers and patient populations.

Furthermore, the asymmetric funnel plot pointed to significant bias, suggested heterogeneity among selected studies and evidence of small-study effects, including selection bias, for example, inclusion criteria of patients undergoing CT imaging based on clinical suspicion rather than systematic screening for ILD. These factors necessitate caution in interpreting the overall pooled prevalence in this study. On the other hand, estimates derived from low-bias and multimodal diagnostic studies (∼10–13%) may provide a more clinically meaningful approximation, though they remain informed estimates rather than definitive prevalence rates. This study definitively underscored the significance of HRCT in the diagnosis of RA-ILD, given that HRCT of the lung is more effective in identifying RA patients with ILD compared with PFTs or chest X-rays. Therefore, uniformly, standardized screening strategy for RA-ILD should be established and prevailing across the world to better understand the actual prevalence of ILD in patients with RA in the clinical setting.

Screening guidelines for patients with systemic autoimmune rheumatic diseases (SARDs)/CTDs, including RA, have been developed by the ACR/American College of Chest Physicians (CHEST), the European Respiratory Society (ERS)/the EULAR, and the Japanese Respiratory Society (JRS)/Japanese College of Rheumatology (JCR) [2–4]. According to the ACR/CHEST guideline, it is not necessary to screen all people with RA, and the panel voted on recommendations for people at higher risk of ILD within each SARD. RA patients with ILD risk factors include high-titer RF/anti-CCP antibodies, cigarette smoking, older age at RA onset, high disease activity, male sex, and higher body mass index. In terms of screening modality, the combination of PTFs and HRCT is preferred over PFTs alone, providing complementary information about the presence and pattern of ILD on HRCT and the physiologic impact evaluated by PFTs.

The ERS/EULAR clinical practice guideline states that patients with RA at risk for ILD could be screened for ILD using HRCT. This recommendation is conditional due to the low certainty of evidence and the lower overall prevalence of ILD in RA. However, not screening at-risk RA patients with HRCT could lead to missing a significant number of ILD cases, which may increase morbidity and mortality. In addition, assessment of respiratory symptoms should be considered. In case of the symptoms or CT abnormalities, conducting PFTs should be considered.

According to the guide for diagnosis of CTD-ILD provided by JRS/JCR, the screening management of ILD involves a risk assessment for developing ILD, followed by making a diagnosis of ILD using screening tools, with subsequent multidisciplinary discussion. This guideline stated that the risk factors for ILD in patients with CTD include male, elderly at disease onset, smoking history, and ILD-related disease-specific antibodies (RF/anti-CCP for RA). Persistent respiratory symptoms (dry cough, palpitation during exercise, shortness of breath), chest auscultation, chest X-ray, HRCT, and measurement of Krebs von den Lungen-6 are considered as the comprehensive screening tools for detecting ILD.

Therefore, HRCT and PTFs are key tools for screening ILD in RA patients based on the statements provided by each regional guideline. On the other hand, it remained unknown how frequently patients with RA should be screened for ILD if ILD is absent at the first screening evaluation due to a lack of evidence for RA [3].

The American Thoracic Society (ATS) provided an approach to the evaluation and management of interstitial lung abnormalities (ILA) [5]. Adults with CTD should undergo chest CT screening to identify ILA/ILD. ILA is defined as nondependent bilateral parenchymal abnormalities on chest CT, including ground-glass opacities, reticular abnormalities, lung distortion, traction bronchiectasis, and/or honeycombing involving >5% of a lung zone. The updated definition removes the prior exclusion of high-risk populations, including CTDs. ILD is distinguished from ILA by symptoms (dyspnoea/cough) attributable to an interstitial process; abnormal or declining lung function; fibrotic (honeycombing and/or reticulation with traction bronchiectasis involving ≥5% of total lung volume) or progressive imaging abnormalities; and/or specific fibrotic ILD patterns on imaging or pathology. Thus, patients with ILA undergo baseline symptom assessment, which is defined as enquiring about the presence of cough and dyspnoea on exertion with high value on non-invasive early identification of ILD. In addition, PFTs should be recommended when ILA is detected.

According to the ILA follow-up method provided by ATS, the frequency of reassessment with CT and PFTs was considered at low-risk populations every 2–3 years, while being conducted at high-risk populations, such as family history of pulmonary fibrosis, older age, smoking history, CTD, fibrotic lesions on HRCT, and abnormal or borderline PFTs, at 12 and 6–12 months, respectively. This concept might be useful for early detection of developing ILD in ILA patients with CTD, including RA.

In summary standardized screening methodology and established diagnostic criteria of ILA/ILD contribute to appropriate management of RA, including early detection of ILA/ILD, prevention of developing ILD, and early intervention of treatment for ILD. We need to conduct further study to evaluate the prevalence of ILA/ILD and deterioration of ILD during follow-up using a prospective inception cohort including incident cases of RA.

## Data Availability

No new data were generated or analysed in support of this article.
